# Medicinal Plants with Potential Inhibitory Bioactive Compounds against Coronaviruses

**DOI:** 10.34172/apb.2022.003

**Published:** 2021-01-30

**Authors:** Olutayo Ademola Adeleye, Oluyemisi Adebowale Bamiro, Lateef Gbenga Bakre, Florence Olubola Odeleye, Modupe Nofisat Adebowale, Olufemi Lionel Okunye, Mariam Adeola Sodeinde, Adannaya Charity Adebona, Farid Menaa

**Affiliations:** ^1^Department of Pharmaceutics and Pharmaceutical Technology, Federal University Oye-Ekiti, Ekiti State, Nigeria.; ^2^Department of Pharmaceutics and Pharmaceutical Technology, Olabisi Onabanjo University, Ago Iwoye, Ogun State, Nigeria.; ^3^Department of Pharmaceutical Microbiology,Olabisi Onabanjo University, Ago Iwoye, Ogun State, Nigeria.; ^4^Department of Pharmacognosy Olabisi Onabanjo University, Ago Iwoye, Ogun State, Nigeria.; ^5^Department of Translational Nanomedicine and Advanced Technologies California Innovations Corporation, San Diego, La Jolla, California, USA.

**Keywords:** Medicinal plant, Bioactive compounds, Coronavirus, SARS-CoV

## Abstract

Medicinal plant is a major source of drug discovery for disease management. Over 85% of the population in Asia and in the Middle East use herbal medicine for disease management such as severe acute respiratory syndrome (SARS) caused by coronavirus. Infection from coronavirus is initiated by entry of the virus into a susceptible host cell. The two human coronaviruses of public health importance two decades ago were SARS-CoV and Middle East respiratory syndrome coronavirus (MERS-CoV) and now SARS-CoV-2. These three viruses belong to the same class of beta coronavirus and are somewhat similar in genome sequencing, life cycle, mode of entry into a host, mode of transmission and clinical manifestations. This review identified twenty medicinal plants with potential inhibitory bioactive compounds from natural sources that are active against coronaviruses that could be developed into various drug delivery systems. It also highlighted several evidences to show that medicinal plant used in the treatment of SARSCoV may offer some sort of relief from the burden of coronavirus disease 2019 (COVID-19) pandemic. Since there is no specific treatment for COVID-19 yet, the search for medicinal plants with inhibitory bioactive compounds against coronavirus could be the long awaited breakthrough scientists have been searching to change the narratives of COVID-19 pandemic.

## Introduction

Drugs are chemically and/or biologically synthesized. Semi-synthesized drugs (e.g. homatropine) are obtained from natural sources while fully synthesized drugs are chemically amalgamated in the laboratory (e.g. paracetamol, aspirin). Hence, plants contain beneficial bioactive compounds that may be valuable for therapeutic purposes or used as precursors for drugs biosynthesis.^1^ The whole plant, part of the plant (e.g. leaves), exudates or extract of plants represent potential sources of bioactive compounds.^2^ Hence, medicinal plants have been applied since time immemorial and it is important to mention that their use is growing dramatically.^3^ Indeed, medicinal plants remain a major source of drug discovery and play an important role in the management of diseases such as infections.^4-6^ It is also worth noting that over 85% of the population in Africa, Asia and in the Middle East use herbal medicine as first line of treatment.^7^ Interestingly, the Chinese population widely utilizes herbs to control severe acute respiratory syndrome (SARS) caused by coronaviruses (CoVs). CoVs are enveloped single-stranded RNA (ss-RNA) viruses that infect both humans and animals. They are named for the crown-like spikes on their surface, and are classified as alpha, beta, gamma, and delta.^8^ They affect the respiratory, gastrointestinal and central nervous systems.^9^ Mechanistically, the CoVs-mediated infection is initiated by the entry of the virus into host cells through binding of the viral spike protein (S-protein) to angiotensin-converting enzyme 2 (ACE2) present in the host (mainly epithelial cells such as pneumocytes and enterocytes). Their replication is controlled by the viral 3-chymotrypsin-like cysteine protease (3CLpro), papain-like proteinase (PLpro) and RNA-dependent RNA protease (RdRp) enzyme.^10,11^ Onset of CoVs symptoms occurs within 14 days of infection and decreases thereafter.^12,13^ Transmission appears to spread mainly through respiratory droplets and contact routes.^12^ The two types of human coronaviruses of public health concerns two decades ago were SARS-CoV and Middle East respiratory syndrome coronavirus (MERS-CoV) until now the recent outbreak of another CoV known as SARS-CoV-2. These three viruses belong to the genera, beta coronavirus.^14^

Human coronaviruses were isolated nearly 50 years ago but they were only thought to cause mild, self-limiting respiratory disease.^15-17^ The outbreak of SARS-CoV changed this thought and gave scientist a new concept and approach to the CoVs. Since then, CoVs have been established to be endemic in the human populations, causing 15–30% of respiratory disease each year.^18^ They cause more severe disease in children below age 5, the elderly, and individuals with underlying chronic illnesses (e.g. cardio-vascular diseases, diabetes etc).

SARS-CoV-2 was discovered in Wuhan, Hubei, China in December 2019. This virus, of unclear origin, is highly virulent, infectious, contagious and lethal. In less than a month, SARS-CoV-2 known as coronavirus disease 2019 (COVID-19) has spread from people to people to other provinces in China and reach many other countries within three months.^19^ As estimated on April, 18^th^ 2020, there were 2 160 207 confirmed cases and 146 088 deaths globally from the pandemic.^20^

At present, the management of symptomatic patients with COVID-19 mainly involves the use of antiviral, supportive treatment (e.g. corticosteroid and mechanical ventilation) while boosting the patient’s immunity. To date, no chemical therapy is officially considered as successful in terms of efficiency and safety.

This review is carried out at this time as the inhibition of SARS-CoV-2 has defied specific therapeutic intervention. It reviews recent advances in the use of medicinal plants for the supportive treatment and cure of COVID-19. Some medicinal plants that possess bioactive compounds with CoV inhibitory activities that could be applied to prevent, treat, or used as adjuvant therapies in the management of COVID-19 are presented.

### Andrographis paniculata

*Andrographis paniculata* (Burm.f.) Nees (*A. paniculata*) is a medicinal plant which belongs to the family, Acanthaceae. It is commonly known as the “King of Bitters”.It is widely distributed and used traditionally in China and tropical Asian region in the treatment of infectious diseases, common cold and upper respiratory tract infection, inflammation, fevers, cancer etc.^21,22^ The main bioactive compound of *A. paniculata* is andrographolide a diterpenoid which has a wide spectrum of antiviral activity among other antimicrobial activities.^23^ There are other bioactive antiviral compounds, such as andrographiside and andrograpanin isolated from this plant.^24^ Some studies indicate that *A. paniculata* possesses inhibitory bioactive compounds against CoVs.^24,25^ Wu et al. deduced that andrographolide and its derivative could serve as new lead compound in drug discovery for the treatment of SARS-CoV-2 infections by inhibiting viral 3CLpro and PLpro enzyme.^24^ Also, andrographolide sulphonate is the main active ingredient of “Xiyanping”, a traditional Chinese antiviral and anti-inflammatorymedicine, used as an injection.^25,26^ Itwas used in the treatment of SARS-CoV infection in 2002 as adjuvant therapy with a significant outcome.^27^

### Rheum species

Rhubarb is the common name of a perennial plant belonging to the genus ‘*Rheum’* in the family*Polygonaceae*. There are several species of this plant e.g. *Rheum emodi* Wall.*, Rheum palmatum L., Rheum officinale*Baill*., Rheum tanguticum* Maxim. ex Balf. They are known to exert some antiviral activities against CoVs.^28,29^ The most important phytoconstituent of the root extract that possesses this antiviral activity is emodin, an anthraquinone derivative. Ho et al. and Schwarz et al. reported that emodin acts by blocking the binding of SARS-CoV S-protein to the host receptor ACE 2, and suggested that emodin could be considered as a potential lead therapeutic agent for SARS.^28,30^

### Polygonum multiflorum

*Polygonum multiflorum* Thunb. is a perennial plant belonging to the genus *polygonum*in the family *Polygonaceae*. It is commonly known as tuber fleece flower, and represents a popular traditional Chinese medicine (TCM) listed in the Chinese Pharmacopoeia (CP).^31^ Laboratory investigations of various parts of this plant demonstrated that *Polygonum multiflorum* displays various bioactive components with antibacterial, anti-inflammatory, anti-oxidant and antiviral properties.^32-34^ Like in rhubarb, the most important bioactive compound of this plant is emodin, which was found to exerts inhibitory activity against SARS-CoV.^28^

### Glycyrrhiza glabra

The root of *Glycyrrhiza glabra* L. is popularly called liquorice. The plant is an herbaceous perennial legume belonging to the family *Leguminosae* and widely distributed in Europe and Asia. The most active bioactive compound is glycyrrhizin which acts by inhibiting the interaction between S-protein and ACE2 thereby preventing viral invasion.^35,36^ Pilcher and Cinatl et al suggested that glycyrrhizin should be assessed for the treatment of SARS as an alternative option.^37,38^ Further investigation was done by Hoever et al and Fiore et al who confirmed the activity of glycyrrhizin against SARS coronavirus.^39,40^

### Scutellariabaicalensis

*Scutellariabaicalensis* Georgi, or Chinese skullcap, is a flowering plant belonging to the family *Lamiaceae*.^41^ It is found abundantly in several East Asian countries and Russia. This plant has been widely used as a medicinal plant in China for thousands of years and has been officially listed in the CP as a medicine to treat various conditions like hepatitis, inflammation, diarrhea, dysentery, hypertension, and respiratory infections.^42^ The biologically active phytoconstituent of the root extract is baicalin, a flavone glycoside known to have anti-inflammatory, anti-allergic, free radical scavenging and apoptotic activities.^43^ This flavonoid can be found in other species from the genus *Scutellaria* such as *Scutellariaamoena* and *Scutellaria likiangensis*.^44^ Interestingly, baicalin is the most active flavonoid from *Scutellaria* spp. against virus.^45^ Wong and Yuen reported baicalin as an antiviral compound with an unknown mechanism that can be used for the management of CoVs with particular reference to SARS.^46^ More recently, Yang et al^36^ reported that baicalin acts by inhibition of ACE 2, making it a valuable antiviral compound in the treatment of patients with SARS-CoV-2.

### Quercetin yielding medicinal plants

Quercetin is a bioactive flavonoid present in many plants and some fruits such as green tea (*Camellia sinensis)*, onions (*Allium cepa*), and apples *(Malus domestica*).^47,48^ It was used in folk medicine as an antioxidant. Most recently, Smith and Smith reported that quercetin^49^ is a FDA-approved compound that prevents CoVs from binding to host cell’s ACE2 receptor. However, Nguyen et al. previously reported that quercetin and some other flavonoids in this class act on CoVs by 3CLpro inhibition,^50^ and this report was substantiated by a most recent study made by Jo et al.^51^ Other flavonoids, such as herbacetin, hesperetin, rhoifolin, pectolinarin, have been proved to possess antiviral activities against CoVs.^52,53^ Some medicinal plants containing these flavonoids include *Toona sinensis* (Juss.) M.Roem. (family - Meliaceae),^54,55^ *Litchi chinensis* Sonn (family - Sapindaceae),^56^ *Pichia pastoris* (family - Saccharomycetaceae),^50^ *Houttuynia cordata*Thunb. (family - Saururaceae),^57^ *Sambucus nigra*L. (family - Adoxaceae).^58^

### Galla Chinensis

The galls on the leaves of *Rhus chinensis* Mill. is known as *Galla Chinensis. Rhus chinensis*is a deciduous flowering shrub belonging to the family *Anacardiaceae* and widely distributed in Asia and commonly known as sumac. *Galla Chinensis* has been used in TCM for several years in the treatment of diarrhea and prolonged coughing.^31^ Djakpo and Yao reported that *Galla Chinensis* contains strong antiviral properties.^59^ The bioactive compound of *Galla Chinensis* that possesses antiviral activity is the polyphenol Tetra-*O*-galloyl-β-d-glucose (TGG), a tannin. Indeed, Ling et al. discovered that TGG is effective against SARS-CoV with a mechanism that would interfere with the virus entry into host cells.^60^

### Phyllanthus emblica

*Phyllanthus emblica* L.is an Indian gooseberry originated from a deciduous flowering tree belonging to the family *Phyllanthaceae* and used in traditional medicines in India to treat cough, constipation, fever and asthma.^61^ This plant possesses various bioactive compounds such as emblicanin, phyllaemblicin, punigluconoin and glochicoccin.^62-64^ Among them, the polyphenol, phyllaemblicin B, an ellagitannin, represents the bioactive compound that has been extensively studied for antiviral activity.^62,65^ Actually, a recent review highlighted phyllaemblicin B as a potential bioactive compound for the treatment of SARS-CoV-2 by inhibiting RdRp.^24^ Like in *Galla Chinensis,* TGG was also found eliciting a good antiviral property in *Phyllanthus emblica*.^66^

### Isatisindigotica

*Isatisindigotica* L. is a small flowering plant with a decumbent stem belonging to the family *Brassicaceae*. It is native to east and central Asia. The dried root of *Isatisindigotica* is commonly called Ban Lan Gen, Woad root, Indigo wood, or *Isatidis Radix*. Woad root has been used in the treatment of cold, headache, sore throat, bacterial and viral diseases for several years in China.^67,68^ During the SARS-CoV and MERS pandemic which occurred in 2003 and 2012, respectively, *Isatisindigotica* was used in the management of people with the said disease.^69^ The antiviral activity of *Isatisindigotica* extractwas confirmed by Hsuan et al, Yang et al, and Ping et al.^70-72^ Interestingly, Yang et al and Su et al concluded that the antiviral activity of *Isatisindigotica* extract is mediated by inhibition of virus attachment.^71,73^ The important bioactive compounds of this plant with antiviral activity are clemastanin B, epigoitrin, sinigrin, indigo, indirubin, beta-sitosterol.^69,72-74^ To date, beta-sitosterol, sinigrin and indigo were shown to be active against CoVs by inhibiting 3CLpro.^36,69,75^

### Erigeron breviscapus

*Erigeron breviscapus* (Vaniot) Handel-Mazzetti is a perennial Chinese flowering herb in the family *Compositae* and its common name is fleabane. It has been used for many years in TCM. Scutellarin is the most active bioactive compound of this plant having anti-inflammatory, anti-oxidative, anti-platelet, and anti-coagulation properties.^76,77^ It is clinically used to treat diabetes and stroke.^78^ Yu et al. observed that scutellarin potently inhibited the activity of SARS-CoV protease enzyme *in vitro* and concluded that it could be a potential SARS-CoV inhibitor.^79^

### Torreya nucifera

*Torreya nucifera* (L.) Siebold & Zucc.is a large evergreen shrub belonging to the family *Taxaceae*. It is commonly known as Japanese nutmeg and it is native to Japan and South Korea. The seed of this plant is used as an anthelmintic to treat several worm infestations. The plant is used to relief pain, and as a carminative, digestive and laxative. The bioactive component of the plant is Amentoflavone, a polyphenolic compound that is also present in many other plant families such as *Selaginellaceae*, *Euphorbiaceae*, *Cupressaceae*, *Calophyllaceae* and *Podocarpaceae*.^80^ So many studies on amentoflavone have proved its pharmacological potentials as antioxidant, anti-inflammatory, antifungal, and antivirus^81,82^ In a study carried out by Ryu et al., it was discovered that among the twelve compounds isolated from *Torreya nucifera*, amentoflavone showed potent activity on SARS-CoV by a molecular mechanism involving the inhibition of 3CLpro.^83^

### Strobilanthes cusia

*Strobilanthes cusia* (Nees) Kuntzeis an herbaceous perennial plant, a member of the family *Acanthaceae*, widely distributed in Asia.^84^ It has no common name associated with it but in some Asian cities, it is called Assam indigo. The root and leaf extracts have been widely used in traditional herbal medicine thanks to its anti-inflammatory, antipyretic, antitumor, antimicrobial, and antiviral properties.^85,86^ The leaf and root extract possesses several bioactive compounds which include aurantiamide acetate, β-sitosterol, indirubin, tryptanthrin, betulin, indigodole B.^87-89^ These compounds exert antiviral activity against various viral infections.^88,90^ Tsai et al studied the antiviral activity of the bioactive compounds of the leaf extract of *Strobilanthes cusia* on human CoV-NL63.^91^ They observed that among the compounds isolated, tryptanthrin displayed the strongest antiviral activity with significant reduction in human CoVs. The suggested mechanism of action of tryptanthrin is the moderation of viral RNA genome synthesis by its activity on viral enzymes like RNA-dependent RNA polymerase and PLpro that are responsible for the late stages of CoV-NL63 replication.^91^

### Veronica linariifolia

*Veronica linariifolia* Pall. ex Link. is a weed commonly called speedwell which belongs to the family*Plantaginaceae*. There are several species of this genus including *V. persica, V. liwanensis, V. filiformis, V. longifolia, V. fuhsi* and *V. peregrine*. They are widely distributed in Australia, New Zealand, New Guinea and western Asia. This plant is used in traditional medicine for wound healing, and rheumatism, among various other diseases. Its various bioactive compounds possess a wide spectrum of activities like anti-inflammatory, antioxidant, antimicrobial and anticancer.^92-94^ The bioactive compounds of importance that have been isolated and investigated from *Veronica linariifolia* are linariifolioside, luteolin, apigenin, vanillic acid, protocatechuic acid, isoerulic acid and catechol.^95,96^ Luteolin is the most active of these compounds against viruses. It is a flavonoid active against SARS-CoV. Although Jo et al revealed that the anti-SARS CoV activity is mediated by inhibition of 3CLpro,^51^ some other studies reported that this activity is exerted through inhibition of S-protein binding with ACE2.^38,60^

### Camellia sinensis

*Camellia sinensis* (L.) Kuntze is an evergreen flowering small tree in the family *Theaceae*. It is commonly known as green tea. Green tea has many health benefits and has been in use for centuries for the treatment of several diseases and conditions, including vomiting, diarrhea, inflammation, infections, Parkinson’s disease, and cancers. Its use in the treatment of viral infection was reported in several studies.^97-99^ The bioactive component of *Camellia sinensis* that is active against CoVs is theaflavin, a polyphenolic compound. Yu et al and Chen et al reported that theaflavin from *Camellia sinensis* has a good anti-SARS CoV activity which is mediated by the inhibition of 3CLpro and RdRp.^79,100^ This observation was supported by a more recent study by Wu et al.^24^

### Swertia kouitchensis

*Swertia kouitchensis* Franch. is a perennial plant distributed mainly in Southern China. It belongs to the family *Gentianaceae* and has no common names associated with it. It is widely used in traditional medicine to treat sore throat, indigestion and jaundice. The plant possesses several bioactive compounds, including xanthones, flavonoids, triterpenoids, alkaloids, which have been shown to be effective as anti-oxidant, antibacterial, antifungal, and antiviral.^98,101,102^ The bioactive compounds that inhibit 3CLpro, making them valuable against CoVs, are Kouitchenside and Oleanolic acid.^24^ Other species in *Swertia* genus with potential anti-SARS CoV activity include* Swertia binchuanensis, Swertia macrosperma, Swertia maculate, Swertia mussotii*.

### Bupleurum spp

*Bupleurum* spp., also known as Saiko or Chai Hu, are annual or perennial herbs or shrubs of a large genus in the family *Apiaceae* native to North America and Southern Africa, and widely distributed in Asia. Some of the important species, that have been used in traditional medicine especially in China, Korea and Japan to treat fever, flu, cough, headache, asthma, chest pain, constipation, diarrhea, epilepsy, fatigue, and/or hepatitis, are *Bupleurum chinense*DC*, Bupleurum scorzoneerifolium, Bupleurum kaoi, Bupleurum falcatum*.^103^ The main bioactive component of this plant is saikosaponin (a triterpene glycoside) which has been found to possess anti-cancer, antiviral, anti-inflammatory, antipyretic, antihepatotoxic, anti-allergic, immunoregulation, and neuroregulation activities.^104,105^ Saikosaponin is also present in some medicinal plants such as *Heteromorpha* spp., and *Scrophularia scorodonia*.^106^ Many studies have reported the antiviral activity of saikosaponin against CoVs by inhibition of viral attachment.^106-108^

### Alnus japonica

*Alnus japonica* (Thunb.) Steud., known as Japanese alder, is a deciduous tree, a member of the family *Betulaceae*. It is found in Japan, Korea, Taiwan, eastern China, and Russia. The genus* ‘Alnus’* iswell-known in Korean folk medicine and in Ayurvedic medicine for the treatment of hepatitis, mouth and throat inflammations, dysentery, stomach ache, diarrhea, fever, cancer.^109,110^ The dominant biologically active natural compounds of *Alnus japonica* is the diarylheptanoids.^111^ These secondary metabolites have also been isolated from other medicinal plants such as *Curcuma kwangsiensis, Alpinia officinarum, Zingiber mekongense, Acer nikoense, Aframomummelegueta, Alpinia katsumadai, Acer nikoense*.^112^ Diarylheptanoids exhibit anti-inflammatory, cytotoxic, antiviral and anticancer activities.^113,114^ Remarkably, diarylheptanoids from *Alnus japonica* have been found to be effective against SARS-CoV by inhibiting PLpro.^111^ Zang & Liu concluded that diarylheptanoids with other natural compounds could be used as alternative choices to fight SARS-COV-2.^115^

### Lonicera japonica

*Lonicera japonica* Thunb. also known as honeysuckleis a deciduous climber belonging to the family *Caprifoliaceae* native to eastern Asia. It is used widely in TCM and contained over 500 prescriptions listed in CP to treat conditions such as cough, cold, tonsillitis, fever, inflammation, pneumonia.^31^ The plant extract consist of several constituents including organic acids and flavones that confers its wide pharmacological activities, such as hepatoprotective, anti-inflammatory, antioxidative, antibacterial, and antiviral activities.^116^ The primary bioactive compound of the plant is chlorogenic acid, a phenolic acid that is active against viruses and other microorganisms.^117^ *Lonicera japonica* has been extensively used to prevent and treat SARS-CoV by inhibiting RdRp involved in SARS-CoV replication.^118,119^ Indeed, it was the most popular plant used in the treatment of SARS epidemic of 2003 in China.^116^ Also, “*Shuang Huang Lian”,* a TCM prescription,^120^ containing* Lonicera japonica*, exerted anti-SARS-CoV-2 activity.^36^

### Aesculus chinensis

*Aesculus chinensis* Bunge, commonly called Chinese horse chestnut, abundantly distributed in northwestern China, is a deciduous tree species in the genus ‘Aesculus’ and a member of the *Sapindaceae* family. The seeds of this tree have been frequently used in folk medicine to treat chest and abdominal pain.^121^ The bioactive constituent of *Aesculus chinensis* is escin, a triterpenoid saponin, which exerts pharmacological activities such as anti-inflammatory, antioxidative, anti-tumor and antimicrobial activity.^122^ Escin was reported to possess antiviral activity against SARS-CoV by inhibiting viral 3CLpro.^118,123^

### Saposhnikovia divaricata

*Saposhnikovia divaricata* (Turcz.) Schischk., commonly called Siler, is an herbaceous perennial plant that belongs to the family *Apiaceae*. The plant is native to China, Russia, Korea and Japan, and is used as herbal medicine for the treatment of general body pain, headaches, spasm, tremor, arthritis and inflammation.^124,125^ The plant possesses several bioactive compounds including coumarins, chromones, lignans, sterols that elicit antioxidative, antimicrobial, anti-inflammatory, immunoregulatory, anti-proliferative, and analgesic activities.^126,127^ The compound that is effective against viruses are the coumarins.^126,128^ Coumarins from the root of *Saposhnikoviae divaricate* is one of the constituents of a TCM formula, named “Yuping feng” powder used to prevent SARS-CoV.^129^ Liu et al showed that *Saposhnikovia divaricate* is contained in some of the Chinese herbal prescriptions recommended by the Chinese government in 2004 for the treatment of SARS-CoV infection.^130^ Further, Yang et al listed it as one of the frequently used medicinal herbs in the prevention of COVID-19 infection.^36^ Also, *Saposhnikovia divaricata* has been shown to be effective against porcine epidemic diarrhea virus, which belongs to the same class (*Coronaviridae*) with SARS and MERS viruses, by a molecular mechanism involving the inhibition of S-protein.^118,128^

## Discussion and Conclusion

Undeniably, plants are great reservoir of compounds valuable for the treatment of infections and other disease conditions.^131^ Importantly, medicinal plants offer some advantages, such as ease of accessibility and availability, as well as low toxicity over synthetic drugs. Twenty medicinal plants with various bioactive compounds that possess potentials for inhibition of SARS-CoV-2 were identified in this review (Table 1). The phytochemical class among these bioactive compounds varied from diterpenoid, anthraquinones, saponin, flavonoid, tannin, alkaloids and steroids, with flavonoids being the prominent class. The mechanism of the antiviral action of these compounds varies, but involved the inhibition of at least one enzyme associated with the coronavirus pathogenesis, i.e. S-protein, which initiates the entry of the virus by binding to host ACE2,^132^ 3CLpro and PLpro, which are both responsible for the cleavage of the polyprotein translated from the viral RNA,^10,11,133^ and RdRp, that catalyzes the synthesis of RNA enzyme.^134^ Many researchers have posited that bioactive compounds with inhibitory activity against SARS-CoV may be active against SARS-CoV-2 because of some similarities they share.^135^ Indeed, they are both beta-coronaviruses with similar genome sequencing and life cycle, albeit their origin may differ. The mode of entry into a host, the RNA replication,^132,136^ as well asthe mode of transmission from person-to-person and clinical manifestations of the disease from both viruses are also similar, hence the name SARS-CoV and SARS-CoV-2. Several studies have reported that medicinal plant used in the treatment of SARS-CoV may offer some sort of relief from the burden of COVID-19 pandemic.^115^ None of the isolated compounds from the medicinal plants have been successfully tried clinically for the treatment of COVID-19. However, several drug developments, formulation processes, pilot test and clinical trials are under way.^25,36,137^

**Table 1:** Medicinal plants with potential Anti-SARS CoV bioactive compounds

**No**	**Plant**	**Common name**	**Compound**	**Action (Inhibition)**	**Reference**
1	Andrographis paniculata	king of Bitters	Andrographolide	3CLpro & PLpro	^24,25^
2	Aesculus chinensis	Horse chestnut	Escin	3CLpro	^118,123^
3	Alnus japonica	Japanese alder	Diarylheptanoids	PLpro	^111,115^
4	Bupleurum spp	Saiko	Saikosaponin	S-protein	^107,108^
5	Camellia sinensis	Green tea	Theaflavin	RdRp & 3CLpro	^24,79,100^
6	Erigeron breviscapus	Fleabane	Scutellarin	3CLpro & PLpro	^79^
7	Galla Chinensis	Sumac	Tetra- O-galloyl-β-d-glucose (TGG)	S-protein	^60^
8	Glycyrrhiza glabra	Liquorice	Glycyrrhizin	S-protein	^36,40^
9	Houttuynia cordata	Quercetin	Rainbow plant	3CLpro	^57^
10	Isatis indigotica	Woad root	Beta-sitosterol	3CLpro	^36,69,75^
11	Lonicera japonica	Honeysuckle	Chlorogenic acid	RdRp	^118,119^
12	Phyllanthus emblica	Indian gooseberry	Phyllaemblicin B	RdRp	^24^
13	Polygonum multiflorum	Fleece flower	Emodin	S-protein	^28^
14	Rheum species	Rhubarb	Emodin	S-protein	^28^
15	Saposhnikovia divaricata	Siler	Coumarins	S-protein	^118,128^
16	Scutellaria baicalensis	skullcap	Baicalin	S-Protein	^36^
17	Strobilanthes cusia	Assam indigo	Tryptanthrin	RdRp and PLpro	^91^
18	Swertia kouitchensis	None	Kouitchenside	3CLpro	^24^
19	Torreya nucifera	Japanese nutmeg	Amentoflavone	3CLpro	^83^
20	Veronica linariifolia	Speedwell	Luteolin	S-protein & 3CLpro	^38,60,51^

Since coronavirus pathogenesis involves some specific enzymes at different stages of the development of COVID-19 disease, a combination of plant extracts, pure bioactive phytocompounds, or plants themselves described in this review article, such as *Alnus japonica, Andrographis paniculata, Glycyrrhiza glabra, Lonicera japonica, Saposhnikovia divaricata, and Veronica linariifolia*, may be beneficial and used in clinical trials for the management of COVID-19. However, safety and compatibility of this combination must be ensured.

In conclusion, several evidences have been highlighted in this review indicating that medicinal plant used in the treatment of SARS-CoV could be useful in the management of COVID-19. The search for medicinal plants with inhibitory bioactive compounds that may be developed into various drug delivery systems against coronavirus could be the long awaited breakthrough scientist have been searching to change the narratives of COVID-19 pandemic since there is no effective cure yet.

## Ethical Issues

Not applicable.

## Conflict of Interest

The authors declare no conflicts of interest.
